# Isolation and Structure Analysis of Chitin Obtained from Different Developmental Stages of the Mulberry Silkworm (*Bombyx mori*)

**DOI:** 10.3390/molecules29091914

**Published:** 2024-04-23

**Authors:** Eryk Jędrzejczak, Patrycja Frąckowiak, Teresa Sibillano, Erica Brendler, Cinzia Giannini, Teofil Jesionowski, Marcin Wysokowski

**Affiliations:** 1 Faculty of Chemical Technology, Institute of Chemical Technology and Engineering, Poznan University of Technology, Berdychowo 4, 60-965 Poznan, Polandteofil.jesionowski@put.poznan.pl (T.J.); 2 Instituto Di Cristallografia-Consiglio Nazionale delle Ricerche (IC-CNR), I-70126 Bari, Italy; 3 Institute of Analytical Chemistry, TU Bergakademie Freiberg, Lessingstr. 45, 09599 Freiberg, Germany; erica.brendler@chemie.tu-freiberg.de

**Keywords:** chitin, mulberry silkworm, bombyx mori, WAXS, FTIR, ^13^C NMR

## Abstract

Chitin, a ubiquitous biopolymer, holds paramount scientific and economic significance. Historically, it has been primarily isolated from marine crustaceans. However, the surge in demand for chitin and the burgeoning interest in biopolymers have necessitated the exploration of alternative sources. Among these methods, the mulberry silkworm (*Bombyx mori*) has emerged as a particularly intriguing prospect. To isolate chitin from *Bombyx mori*, a chemical extraction methodology was employed. This process involved a series of meticulously orchestrated steps, including Folch extraction, demineralization, deproteinization, and decolorization. The resultant chitin was subjected to comprehensive analysis utilizing techniques such as attenuated total reflectance–Fourier transform infrared spectroscopy (ATR-FTIR), ^13^C nuclear magnetic resonance (NMR) spectroscopy, and wide-angle X-ray scattering (WAXS). The obtained results allow us to conclude that the *Bombyx mori* represents an attractive alternative source of α-chitin.

## 1. Introduction

Chitin exhibits a broad distribution in nature, rendering it the second most prevalent polymer in natural ecosystems [1,2,3,4,5,6]. Annual biosynthetic production estimates suggest a staggering output ranging from 10^12^ to 10^14^ tons [7]. Predominantly sourced from aquatic organisms, particularly crustaceans like crabs, crayfish, and shrimps [8], chitin also manifests in fungal cell walls [9] and sponge skeletons [10]. However, insects emerge as an increasingly viable and promising alternative source, given that their exoskeletons consist predominantly of chitin [11]. In the realm of insects, the burgeoning accessibility of their biomass and chitin-rich industrial remnants, notably their skeletons and exoskeletons, assumes particular significance [12]. This reservoir of chitin exhibits reduced susceptibility to seasonal fluctuations compared to marine fauna, while their prolific reproductive capabilities render breeding procedures notably more straightforward [13].

Chitin seldom occurs in a pristine state in nature, often coexisting with a plethora of proteins, pigments, and minerals [14]. Structurally, chitin comprises N-acetylglucosamine residues interconnected via β-glycosidic bonds, giving rise to extensive linear chains bolstered by an array of intra- and intermolecular hydrogen bonds. Consequently, this intricate molecular architecture fosters the generation of resilient chitin microfibers [15]. Due to its pervasive hydrogen bonding network and inherent crystalline nature, chitin exhibits minimal reactivity and is insoluble in water, as well as in the majority of organic and inorganic solvents, barring those with the capacity to disrupt the hydrogen bonds network [16,17,18,19,20]. Chitin naturally occurs in one of three polymorphic configurations: α-chitin, β-chitin, or γ-chitin [14]. These variants diverge structurally due to variations in the orientation of the polymer chains. The predominant α-form of chitin exhibits an antiparallel arrangement of adjacent chains, which is responsible for the considerable rigidity of this chitin variety. Conversely, β-chitin features the parallel alignment of chains, while γ-chitin presents a combination of both antiparallel and parallel chain arrangements. Consequently, the properties of γ-chitin fall intermediate to those of α-chitin and β-chitin [19]. Among the polymorphic forms discussed, α-chitin emerges as the most stable and prevalent variety, commonly sourced from sponges [21] and crustaceans [22]. Conversely, mollusks [20] and diatoms [23] serve as primary reservoirs of β-chitin. The γ-chitin variant, on the other hand, predominantly resides within the cocoons of select insect species [24,25].

**Figure 1:**
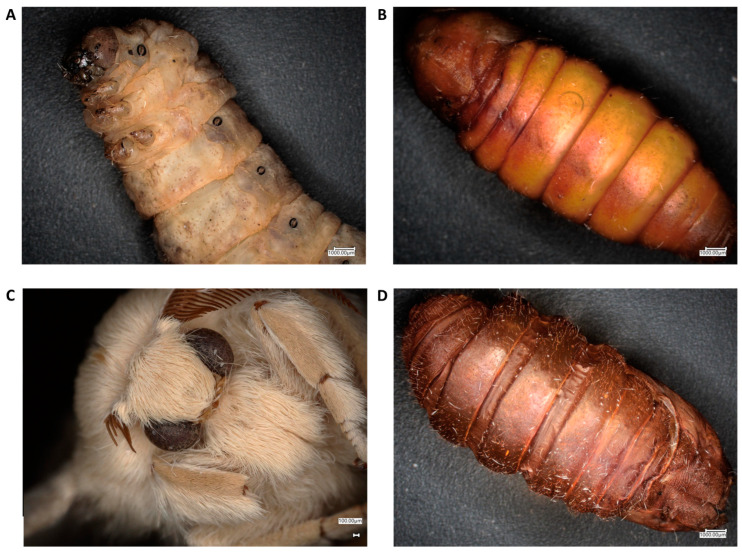
Optical microscope images of mulberry silkworm stages and appendage of the pupa used for chitin extraction, respectively: (**A**) larval stage, (**B**) cocoon (**C**) imago and, additionally, (**D**) remnant of the pupa.

Polymers encompass a diverse array of compounds utilized across multiple domains. However, not all of them exhibit biodegradability or environmental friendliness. In stark contrast, chitin distinguishes itself owing to its extensive natural occurrence, biocompatibility, and minimal toxicity, thereby boasting a broad spectrum of applications. In the contemporary industrial landscape, the management of waste poses a substantial challenge, underscoring the imperative to explore alternative avenues. Notably, insects have garnered considerable attention, particularly in the realm of procuring proteins for the food industry [26]. In the process of protein isolation, by-products such as chitin are typically acquired. The isolation of chitin from insect exoskeletons presents an opportunity to bolster sustainability efforts and mitigate waste accumulation [27]. Additionally, insects have garnered considerable scientific interest due to their reputation as rich sources of diverse compounds, notably proteins and polysaccharides [24,25,28,29,30]. Among these, the mulberry silkworm (*Bombyx mori*) stands out as one of the most extensively studied insects in terms of its potential applications. Silkworms (Figure 1) have been used by people since ancient times to produce silk. This production process has been, and continues to be, remarkably relevant, as evidenced by the dissemination of silk production worldwide [14,15]. Recently, silkworms have also begun to arouse interest as an alternative and interesting source of chitin, leading to a constant publication of works on chitin extraction from different developmental stages of this insect [31,32]. Among the insects frequently cited as sources of chitin and chitosan, noteworthy species include the mulberry silkworm, black soldier fly, housefly, yellow mealworm, superworm, house cricket, field cricket, and desert locust [33]. In terms of the applications of chitin derived from insects and extracted from shrimps, they exhibit little disparity from each other [34]. However, the scientific literature concerning the extraction and utilization of this biopolymer and its derivatives from insects remains scarce [33]. Chitin, upon conversion into its more soluble derivatives, primarily chitosan, demonstrates utility across a wide array of fields [28]. One of the primary domains of application is the food industry, wherein chitin serves as a thickening agent, colour stabilizer, and natural flavour enhancer [33]. Due to its properties such as biocompatibility, biodegradability and low toxicity, chitin finds application in biomedicine, especially for wound dressing and tissue-engineering purposes [35,36]. Significant potential in agricultural applications is facilitated by its antibacterial, fungicidal, and other relevant properties [37]. Additionally, chitin exhibits the capability to bind to a variety of heavy metals such as mercury, lead, zinc, cadmium, chromium, iron, and copper, thereby facilitating their removal from water sources [33]. However, all previously published works have focused solely on isolating and testing chitin from specific developmental stages. Therefore, this is the first work that approaches the subject more comprehensively, using the method of chemical isolation to obtain and compare chitin from all the developmental stages and remnants of the pupa of the mulberry silkworm. Chitins were investigated utilizing attenuated total reflection infrared spectroscopy (ATR-FT-IR), ^13^C nuclear magnetic resonance spectroscopy (NMR), as well as wide-angle X-ray scattering (WAXS) techniques [38,39].

## 2. Results and Discussion

### 2.1. Chitin Content

The chitin content was calculated on the basis of the following formula:

$$\frac{dry\;weight\;of\;the\;sample\;after\;extraction}{dry\;weight\;of\;the\;sample\;before\;exctraction}\;*\;100\%$$

**Figure 2:**
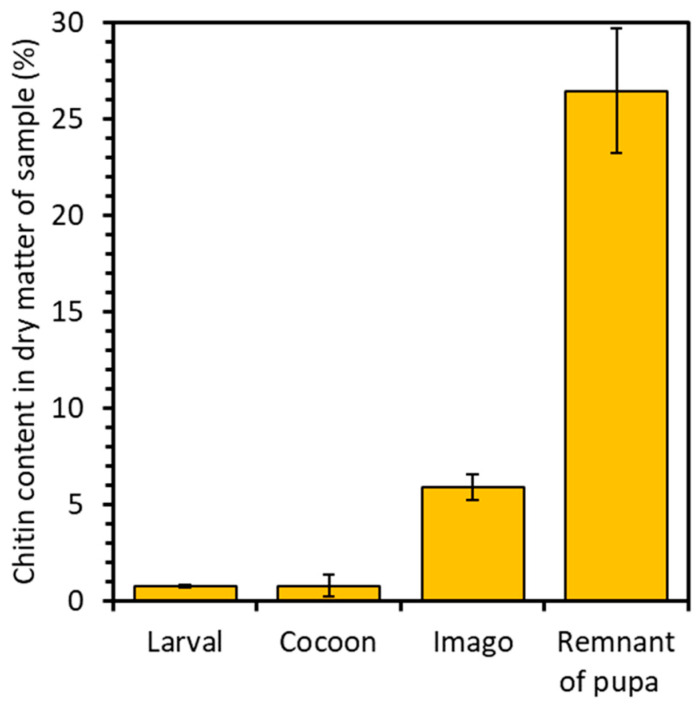
Percentage of chitin in the analyzed samples of silkworm skeletons.

The analysis revealed distinct chitin content variations across the developmental stages and remnant of the pupa of the mulberry silkworm. For the larval stage, 0.7% chitin content was attained, while for the cocoon, it reached 0.8%, and for the imago, it amounted to 5.9% in dry matter. Notably, the pupal residue exhibited the highest chitin content, reaching 26.5% (Figure 2). This significant disparity in chitin levels underscores the pivotal role of chitin as a primary constituent in forming protective shells. These shells act as crucial barriers, safeguarding the delicate internal structures of the pupa from external influences. Consequently, the heightened chitin content within the pupal stage reflects the increased demand for chitin in constructing and reinforcing these vital structures. Such insights illuminate the dynamic interplay between chitin utilization and developmental requirements throughout the silkworm life cycle. A study conducted by P. Battampara et al. [31] demonstrated that employing the processes of deproteinization, decolorization, and demineralization enabled the isolation of 18% chitin content in the dry matter derived from Silkworm pupa. In contrast, the research conducted by A.T. Paulino et al. [40] demonstrates the isolation of 2.59–4.23% chitin from silkworm chrysalides. Comparing these findings with our own results, it is evident that the methodology employed facilitated the extraction of a greater quantity of chitin, with the remnants of the pupae exhibiting the highest chitin content.

### 2.2. Attenuated Total Reflectance—FTIR

**Figure 3:**
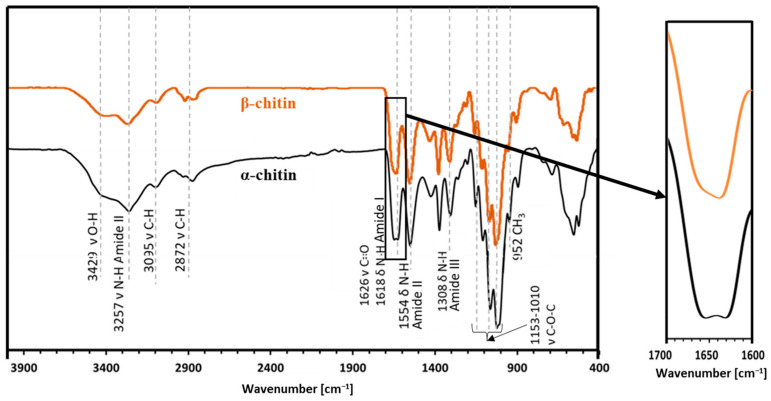
The ATR-FT-IR spectra obtained for the reference sample of α-chitin compared to the reference sample of β-chitin.

**Figure 4:**
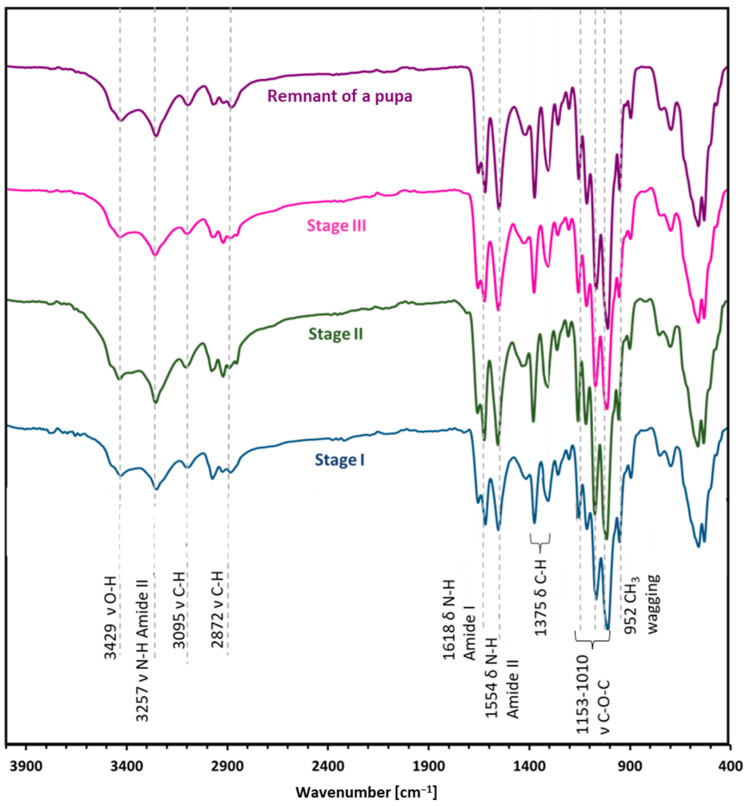
The ATR-FTIR spectra were obtained for the respective developmental stages of the mulberry silkworm: Stage I—larval stage, Stage II—cocoon, Stage III—imago form, and remnant of a pupa.

ATR-FT-IR analysis (Figure 3) makes it possible to determine the chemical structure of the isolated chitin and to determine its polymorphic structure [41]. This analysis utilized α and β chitin standards to discern spectral differences between these variants and compare them with the spectra obtained from the samples. This enables precise identification of whether the isolated chitin from the mulberry silkworm aligns with the α or β variety. For α-chitin, the presence of the amide I band at a wavelength close to 1620 cm^−1^ is characteristic. The wavelength of 1554 cm^−1^ corresponds to that characteristic of the amide band II region, while the band at 1308 cm^−1^ characterizes the amide band III [14,41,42]. The stretching bands observed at 3249 cm^−1^ and 3257 cm^−1^ correspond to the vibrations of O-H and N-H bonds, respectively. The peak at 1375 cm^−1^ represents the C-H bending bond, while a peak at 1626 cm^−1^ indicates the presence of the C=O carbonyl group. The spectrum obtained for the β-chitin variety differs slightly from that of α-chitin, as it is shown in Figure 3. The spectrum of α-chitin shows two signals originating from the amide I band at wavelengths of 1618 cm^−1^ and 1650 cm^−1^. In contrast, the infrared spectrum of β-chitin shows two strongly overlapping peaks in the amide I region, with the peak at 1626 cm^−1^. It is a commonly used feature to distinguish the chitin polymorphs [14,43]. The other bands are at similar wavelengths [41]. The comparison of the spectra of chitin isolated from different stages and the pupal residue of the mulberry silkworm with the spectra obtained for the standards of α- and β-chitin polymorphic variants revealed that the α form is present in all the stages studied and the remnant of the pupa (Figure 4).

### 2.3. Nuclear Magnetic Resonance (NMR)

**Figure 5:**
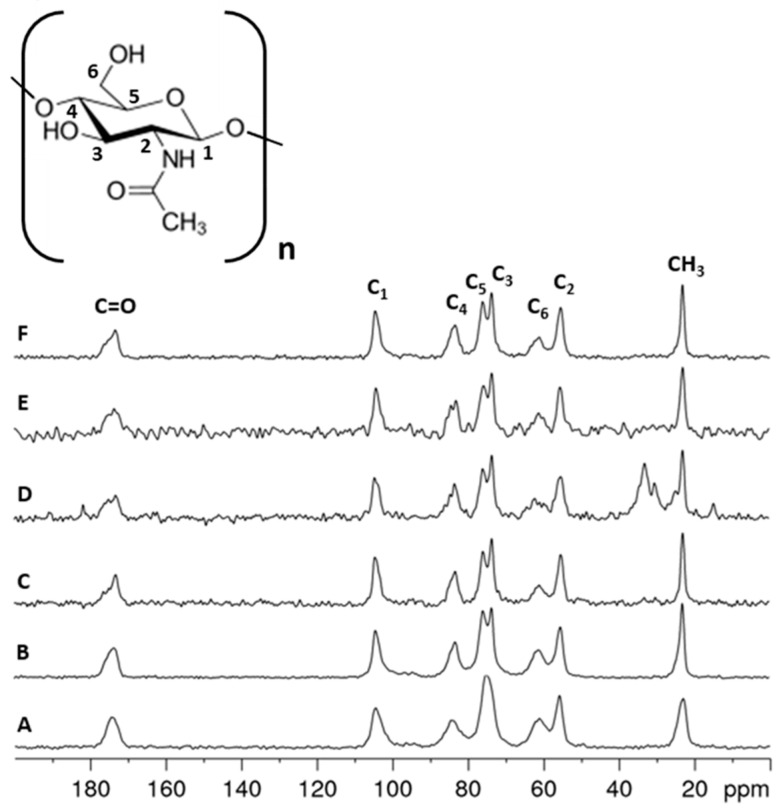
^13^C NMR spectra obtained for the following: (A) β-chitin standard sample, (B) α-chitin standard sample, (C) Stage I—larval stage, (D) Stage II—cocoon, (E) Stage III—imago form and (F) Stage IV—remnant of pupa.

Solid-state ^13^C NMR spectroscopy is a very effective analytical technique for analyzing solid renewable raw materials, such as polysaccharides, including chitin and its derivatives. This technique determines the structure and distinguishes polymorphic varieties. The method makes it possible to analyze solid polysaccharide samples extracted from the different life stages of the mulberry silkworm (*Bombyx mori*) and examine their structure. It also enables the determination of various properties, including the degree of acetylation of the chitin [44]. The ^13^C NMR spectra (Figure 5) recorded for chitin samples isolated from different developmental stages of the mulberry silkworm clearly confirm the α variety in all cases. The main signals for α- and β-chitin varieties are seen in the 110–50 ppm range, with a characteristic shift of the signal of the C2 carbon due to the nitrogen substitution. The differences between the varieties are particularly apparent for the two signals around 75 and 73 ppm, which correspond to the carbon numbers C5 and C3, respectively. For the α-chitin variety, the occurrence of well-resolved peaks for C3 and C5 is characteristic. In contrast, it is typical for β-chitin to have similar chemical shifts for both carbons resulting in a broadened singlet around 74 ppm, which makes it possible to differentiate between the chitin varieties. Such a difference may be due to the fact that the antiparallel and parallel arrangement of the polymer chains in a- and b-chitin, respectively, results in a different hydrogen bridge network for the two modifications. Consequently, the configurations of the C5 and C3 carbon atoms in these chitin varieties are different due to the differences in hydrogen bonding strength [41]. All the spectra of the silkworm samples show the two resolved C3 and C5 resonances characteristic for α-chitin and, thus, confirm the results of the previously discussed methods. In addition, the spectra show that, except for the cocoon sample, which still contains aliphatic-carbon-containing impurities, the isolation of the chitin was successful. A comparison of the signal intensities of the acetamide group and the chitin backbone shows that the degree of acetylation does not change within the different stages of the silkworm evolution.

### 2.4. Wide-Angle X-ray Scattering

**Figure 6:**
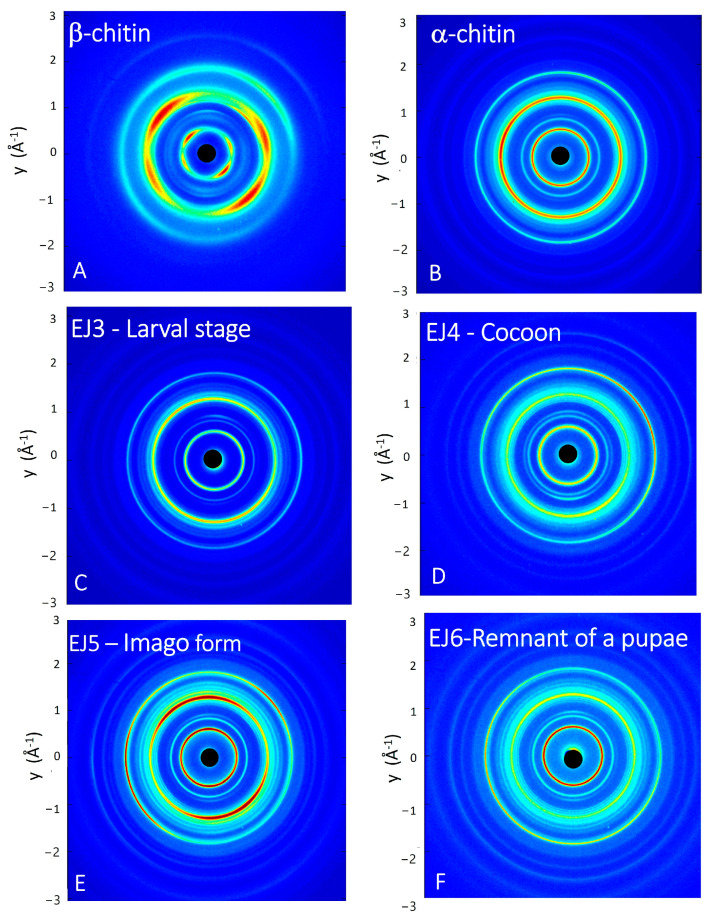
Two-dimensional WAXS patterns of the following: (**A**) β-chitin standard sample, (**B**) α-chitin standard sample, (**C**) larval stage, (**D**) cocoon, (**E**) imago form and (**F**) remnant of a pupa.

The wide-angle X-ray scattering (WAXS) technique permits us to analyze the interference pattern of the secondary waves scattered by the atomic electron density distribution of the chitin crystalline structure. WAXS data allow us to obtain structural information at the atomic scale, thus distinguishing polymorphic crystalline forms, which differ in the packing and polarities of adjacent polymer chains [45]. The as-acquired 2D WAXS patterns (Figure 6A–F) show full diffraction rings, without any preferential orientation for the flakes, as for a randomly oriented powder. This highlights that the native fibrillar structure of chitin [46] has been modified by the process of isolation, since the 2D WAXS patterns do not show the typical fiber pattern with preferential orientation along specific directions [47].

**Figure 7:**
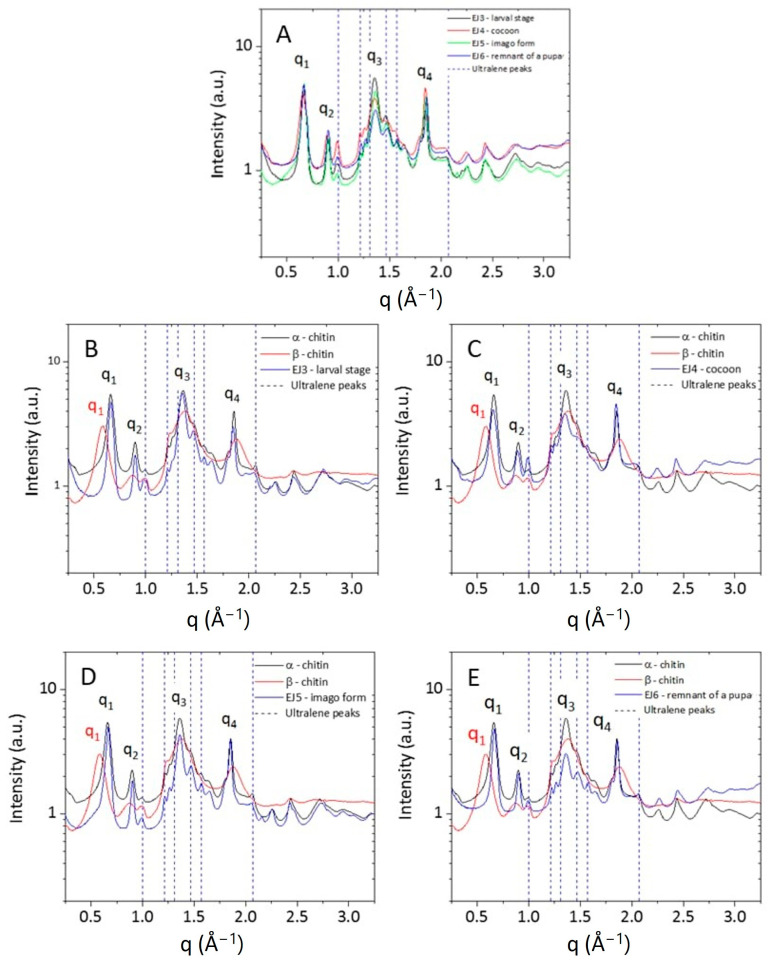
One-dimensional WAXS profiles of (**A**) α- and β-chitin standard samples (red and blue curve, respectively). (**B**–**D**) show a comparison between the 1D profiles of α- and β-chitin reference samples and the ones collected from respective developmental stages (larval, cocoon, imago) and (**E**) remnant of a pupa.

The 2D WAXS patterns, once centred and calibrated, were folded into 1D profiles (Figure 7). The α conformation is one of the most abundant and stable polymorph with the unit polymer chains arranged in an antiparallel configuration and the adjacent chains in the opposite direction. The measured WAXS diffractograms for the α-chitin standard show four sharp reflections at q_1_ = 0.66 Å^−1^ (d_1_ = 9.5 Å), q_2_ = 0.89 Å^−1^ (d_2_ = 7.0 Å), q_3_ = 1.36 Å^−1^ (d_3_ = 4.6 Å) and q_4_ = 1.82 Å^−1^ (d_4_ = 3.4 Å), respectively. These results are in agreement with the literature in which the crystalline lattice structure of the α-chitin was found to be the orthorhombic space group P212121 with unit cell dimensions a = 4.74 Å, b = 18.86 Å and c (fibre axis) = 10.32 Å [46]. This polymorph has a more rigid structure due to the abundance of intersheets and intrasheets.

Compared to α-chitin, the 1D WAXS profile of the β-chitin exhibits a broad diffuse scattering peak and a small shift towards higher distances for the first peak falling at q_1_ = 0.58 Å^−1^ (d_1_ = 10.8 Å). These features correspond to the structure of β-chitin [46], which has a monoclinic unit cell, with a = 4.85 Å, b = 9.26 Å, c = 10.38 Å (fiber axis), and γ = 97.5°. The β polymorph is less frequent in nature and all chains are in the same direction and parallel; in this conformation, the crystallinity is lower because of the parallel arrangement of the polymer chains and because, along the b-axis direction, the short hydroxyl group of one chain is linked by a water molecule to the longer hydroxymethyl group of an adjacent chain. The presence of the water molecule between the two chains allows for more water to enter with an increase in humidity and reduction in crystallinity [48]. The 1D WAXS profiles acquired for the chitin samples extracted from the stages of the mulberry silkworm (Larval stage, Cocoon, Imago and Remnant of a pupa) were compared with 1D WAXS profiles of standard α-chitin and β-chitin. All patterns show a perfect overlap with the WAXS pattern of the α-chitin structure, both for the positions of the diffraction peaks and for their widths, confirming the results obtained by the NMR characterization.

## 3. Materials and Methods

### 3.1. Reagents

**Figure 8:**
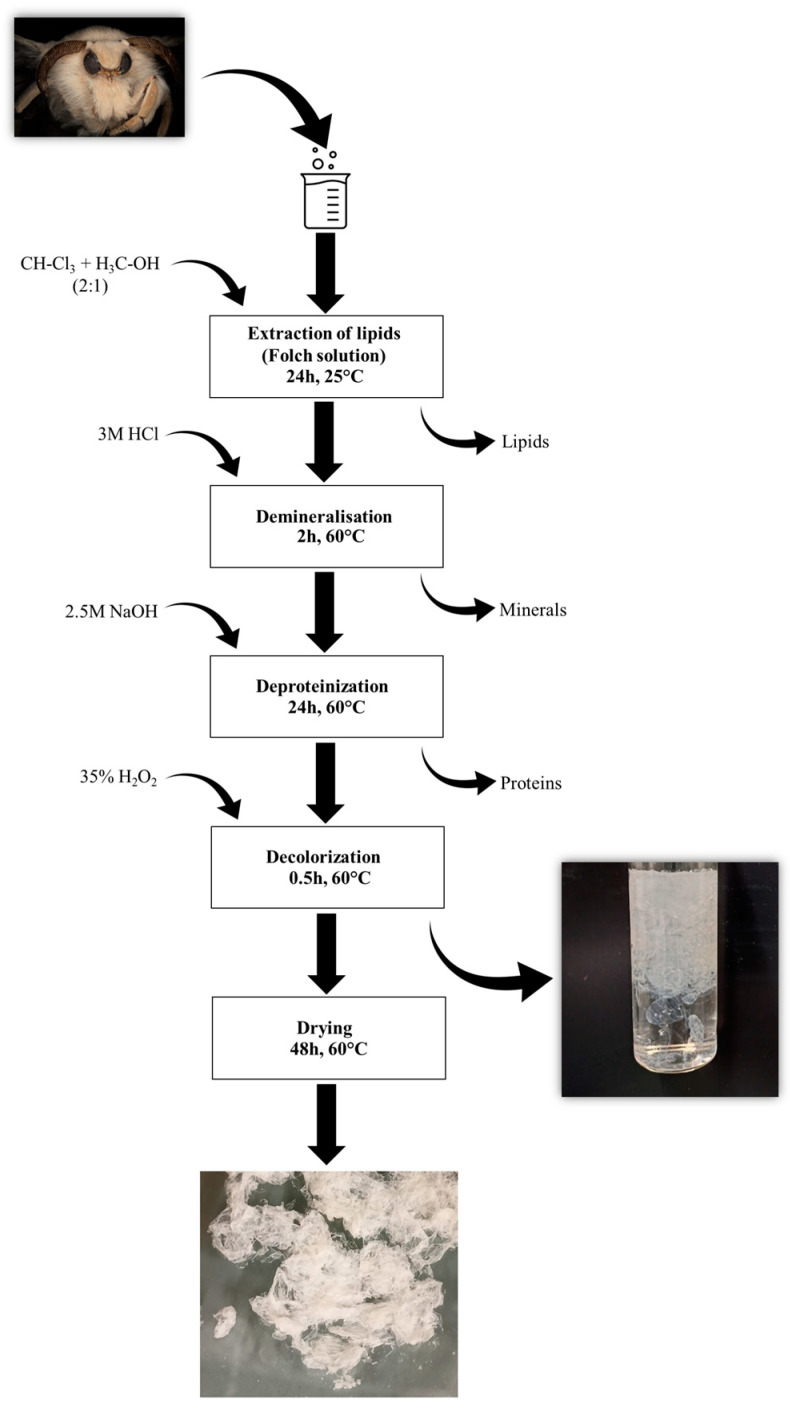
Schematic illustration of the isolation procedure for chitin from mulberry silkworms.

The specific developmental stages of the mulberry silkworm and remnant of the pupa come from the only Polish silkworm farm run by the Institute of Natural Fibers and Medicinal Plants in Poznań, a National Research Institute. The compounds used during chitin extraction (Figure 8), namely chloroform (≥99.9%), methanol (≥99.9%), sodium hydroxide (≥98%), hydrochloric acid (37%), and hydrogen peroxide (35%), were purchased from Merck KgaA (Darmstadt, Germany). Distilled water was used to prepare every solution utilized during the tests.

### 3.2. Instrumentation

ATR-FT-IR analysis was carried out using a VERTEX 70 spectrometer (Bruker, Munich, Germany). Dried skeletons of mulberry silkworms were analyzed for the preliminary identification of the isolated polysaccharide. In addition to the samples from the different life stages of the mulberry silkworm, pure α-chitin and β-chitin were used as standards.

^13^C cross polarization (CP) MAS spectra were recorded on a Bruker AV III 400 MHz spectrometer (Billerica, MA, USA), operating at 400.19 MHz for ^1^H and 100.63 MHz for ^13^C. Spectra were acquired with a 4 mm MAS probe head using ZrO_2_ rotors at a MAS frequency of 8 kHz. A proton 90° pulse length of 4 μs and a 2 ms contact pulse with a 50% ramp were applied. The repetition delay was 4 s and the spectral width 44 kHz. In total, 1024 scans were accumulated with a time domain size of 4 K data points and swftppm decoupling applied during acquisition (AQ = 46 ms).

Wide-angle X-ray scattering (WAXS) measurements were performed at the X-ray Micro Imaging Laboratory (XMI-L@B) of the IC-CNR in Bari, Italy. The experimental setup comprised a Fr-E+ SuperBright copper anode MicroSource (λ = 0.154 nm, 2475 W) which was coupled through a focusing multilayer optics Confocal Max-Flux (Rigaku, Akishima, Japan, CMF 15–105) to a three-pinhole camera for WAXS data collection. The beam size at the sample was shaped down to 0.3 × 0.3 mm^2^. Flakes of standard α- and β-chitin, together with samples of extracted chitin from the stages of the mulberry silkworm (Larval stage, Cocoon, Imago and Remnant of a pupa), were kept in Ultralene^®^ sachets, mounted on a sample holder and placed in a chamber in a vacuum (0.1/1 mbar) for data acquisition. WAXS data were acquired by using an image plate (IP) detector (250 × 160 mm^2^, 100 μm effective pixel size) located at around 10 cm distance from the sample to collect data in a range of 1.8/21 Å in direct space (0.3 to 3.5 Å^−1^ in reciprocal space). WAXS measurements were digitally extracted by an off-line Rigaku RAXIA reader (Rigaku, Akishima, Japan) and elaborated by SAXSGUI v2.05.07 and SUNBIM program. Detector–sample distances were calibrated by means of Si Nist standard powder.

### 3.3. General Procedure

Chitin was isolated using a chemical approach; the schematic view on the isolation procedure is presented in Figure 8. In the first step, Folch extraction was performed to remove the fats and lipids present in the skeletons of mulberry silkworms. Briefly, the insect skeletons were treated with a mixture of chloroform and methanol in a molar ratio of 2:1 for 24 h at room temperature. After the specified time, Folch’s solution was removed, and the degreased skeletons were washed with distilled water and then demineralized. The silkworms’ skeletons were treated with the 3 M HCl solution and left for 2 h at 60 °C. Then, the hydrochloric acid solution was removed, and the silkworm skeletons were washed several times with distilled water to neutralize the pH of the samples. In the next step, skeletons were immersed in a 2.5 M sodium hydroxide solution to remove the proteins associated with the chitin present in the samples. The silkworms were left in this solution for 24 h at 60 °C. The whole process of washing samples with a fresh sodium hydroxide solution was repeated several times under the above-mentioned conditions. The last step of the chitin isolation from insect samples was the decoloration of the isolated polysaccharide. For this purpose, skeletons were treated with 35% hydrogen peroxide for 30 min at 60 °C. After the process was completed, the H_2_O_2_ present in the samples was removed and the silkworms were then washed several times with distilled water. After the extraction process, the obtained polysaccharide was dried at 60 °C for 48 h.

## 4. Conclusions

The method of chitin isolation from mulberry silkworm (*Bombyx mori*) skeletons by chemical extraction turned out to be effective. The obtained values show that the highest content is characterized by the pupa remnant, while the lowest content of chitin occurs in stages I and II (respectively: larval stage and cocoon). Due to the fact that the resulting chitin content is as high as 26%, silkworms are an interesting and alternative source of chitin that contributes to sustainable development. Breeding these insects is simple due to their high reproduction rate, and thus, allows for the acquisition of a significant amount of this polysaccharide. The conducted ATR-FT-IR analysis proves that, in each of the tested samples representing given developmental stages, there is a variant of α-chitin. The FTIR spectra acquired for the studied samples align with the data documented in the literature [41,49]. Studies also indicate the presence of this polysaccharide in the isolated material. ^13^C NMR analysis confirms that the α variant is present in each of the samples. The obtained results coincide with those reported in the literature [50,51]. The WAXS diffractograms show the difference in molecular structure between α- and β-chitin, confirming the different polymorphism in structures. The obtained samples show the same molecular structure of the α-chitin polymorph. In the context of WAXS, the obtained results align with findings documented in publications [52,53]. The analyses above ultimately determined that the chitin present in each of the investigated life stages of the mulberry silkworm and pupal remnants is of the α variety.

## Data Availability

Data are contained within the article.
